# Correlation between Histological Activity and Endoscopic, Clinical, and Serologic Activities in Patients with Ulcerative Colitis

**DOI:** 10.1155/2016/5832051

**Published:** 2015-12-29

**Authors:** Dae Bum Kim, Kang-Moon Lee, Ji Min Lee, Yoon Yung Chung, Hea Jung Sung, Chang Nyol Paik, Woo Chul Chung, Ji-Han Jung, Hyun Joo Choi

**Affiliations:** ^1^Department of Internal Medicine, St. Vincent's Hospital, College of Medicine, The Catholic University of Korea, Seoul 403-720, Republic of Korea; ^2^Department of Hospital Pathology, St. Vincent's Hospital, College of Medicine, The Catholic University of Korea, Seoul 403-720, Republic of Korea

## Abstract

*Objectives*. Recent studies suggest that histological healing is a treatment goal in ulcerative colitis (UC). We aimed to evaluate the correlation between histological activity and clinical, endoscopic, and serologic activities in patients with UC.* Methods*. We retrospectively reviewed medical records from patients with UC who underwent colonoscopy or sigmoidoscopy with biopsies. The Mayo endoscopic subscore was used to assess endoscopic activity. Biopsy specimens were reviewed by two blinded pathologists and scored using the Geboes scoring system.* Results*. We analyzed 154 biopsy specimens from 82 patients with UC. Histological scores exhibited strong correlation with endoscopic subscores (Spearman's rank correlation coefficient *r* = 0.774, *p* < 0.001) and moderate correlation with C-reactive protein levels (*r* = 0.422, *p* < 0.001) and partial Mayo scores (*r* = 0.403, *p* < 0.001). Active histological inflammation (Geboes score ≥ 3.1) was observed in 6% (2 of 33) of the endoscopically normal mucosa samples, 66% (19 of 29) of mild disease samples, and 98% (90 of 92) of moderate-to-severe disease samples.* Conclusions*. Histological activity was closely correlated with the endoscopic, clinical, and serologic UC activities. However, several patients with mild or normal endoscopic findings exhibited histological evidence of inflammation. Therefore, histological assessment may be helpful in evaluating treatment outcomes and determining follow-up strategies.

## 1. Introduction

Ulcerative colitis (UC) is a chronic inflammatory bowel disease that is characterized by a relapsing and remitting course. In patients with UC, it is important to accurately determine the disease activity for assessments and predicting treatment outcomes, and the assessment of UC activity is usually based on a combination of clinical, serologic, and endoscopic findings. Clinical and serologic assessments, which are noninvasive, inexpensive, and easy to use in clinical practice, have typically been used to provide a complimentary measure of the relative degree of inflammation and to monitor disease activity, as no single method has been proven to be ideal in assessing disease activity. Recent studies have suggested that endoscopic assessment of mucosal healing can be used as a measure of disease activity and as a primary end point. In addition, mucosal healing is reported to be associated with sustained clinical remission, decreased need for surgery, decreased hospitalization rates, and reduced risk of colon cancer [1–4]. However, endoscopic findings are not always consistent with histological activity and tend to underestimate the depth of mucosal damage, relative to the histological findings [5, 6].

In contrast, several clinical studies have demonstrated that histological improvements may be associated with better clinical outcomes, including reduced cancer risk and relapse rates; these studies refer to histological remission as a therapeutic goal in UC [7–9]. As with the other assessment modalities, the increased interest in histological assessment has led to the development of several indices that are used to assess histological activity in patients with UC. Among these indices, the Geboes score exhibits good reproducibility and modest agreement with the endoscopic grading system [10].

However, endoscopy and biopsies are expensive and time-consuming and are associated with potential risks. Therefore, it is important to determine the relationship between histological findings and the other disease activity indices for monitoring disease activity and predicting treatment response in UC patients. To our knowledge, there is limited data available regarding these relationships; therefore this study was designed to assess histological activity in patients with UC using the Geboes grading system and to investigate the relationship between the histological activity and the endoscopic, clinical, and serologic activities in these patients.

## 2. Materials and Methods

### 2.1. Study Design and Population

We retrospectively reviewed the medical records of 82 patients with UC who were treated at our hospital between January 2011 and January 2014. During the period, colonoscopies or sigmoidoscopies with biopsies were performed once in 65 patients, twice in 10 patients, three times in 3 patients, and four times in 2 patients. The total number of endoscopies performed in the 82 patients was 102. All patients were diagnosed with UC based on the clinical, endoscopic, and histological criteria [11]. The present study was conducted after obtaining institutional review board approval from St. Vincent's Hospital (VC14RISI0084).

### 2.2. Assessment of Disease Activity

The clinical and endoscopic disease activities were determined using the Mayo scoring system, partial Mayo score (range, 0 to 9) for clinical activity, and Mayo endoscopic subscore for endoscopic activity. The Mayo endoscopic subscore is calculated as inactive disease and normal mucosa (0), mild disease (1), moderate disease (2), or severe disease (3). We performed endoscopy for the assessment of disease activity and therapeutic efficacy or for cancer surveillance. High-definition colonoscopies were used in all examinations (CF-H260AI; Olympus Optical Co., Tokyo, Japan). A blood sample was also drawn near the time of the endoscopy to measure each patient's white blood count, hemoglobin, hematocrit, erythrocyte sedimentation rate, and C-reactive protein (CRP) levels. Abnormal laboratory test results were defined as results that were outside the reference range for our laboratory.

At the time of the colonoscopy or sigmoidoscopy, biopsies were taken from inflamed or healed colonic mucosa. In our routine clinical practice, one or two biopsy specimens targeted at the most inflamed area were taken from the left colon or rectum. If inflamed lesion was not present, the biopsies were collected from random sites of sigmoid colon or rectum. The biopsy specimens were fixed in formalin, embedded in paraffin, and sections were stained with hematoxylin and eosin. The biopsy specimens were then reviewed by two blinded expert pathologists and graded using the Geboes grading system (Table 1) [10]. When the biopsies that were collected displayed various activities, the specimen with the highest grade was assessed. The biopsy specimens were reevaluated for cases showing disagreement between pathologists. Two pathologists reviewed the cases together and reached an agreement for samples with inconclusive results. The Geboes score ranges from 0 to 5.4, with higher scores indicating more severe inflammation, and we defined UC as active histological inflammation with a Geboes score of ≥3.1 [12, 13].

### 2.3. Statistics

All statistical analyses were performed using SPSS software (version 18.0, SPSS Inc., Chicago, IL). Continuous data were expressed as mean ± standard deviation and analyzed using independent sample *t*-tests, whereas categorical variables were expressed as quantities and analyzed using the Mann-Whitney test. Correlations between the variables were estimated using Spearman's rank order correlation coefficient (*r*). *p* values of <0.05 were considered significant for the analysis.

## 3. Results

### 3.1. Patients' Characteristics

The baseline characteristics of the 82 patients are summarized in Table 2. The mean patient age was 47.5 years and the mean duration of disease was 5.4 years. The extent of disease was proctitis in 18 patients (22%), left-sided colitis in 33 patients (40%), and extensive colitis in 31 patients (38%). Seven percent of patients were receiving anti-TNF therapy (infliximab or adalimumab) and 26% of patients were receiving systemic corticosteroids.

### 3.2. Correlation of the Histological Activity with the Endoscopic, Clinical, and Serologic Activities

**Figure 1:**
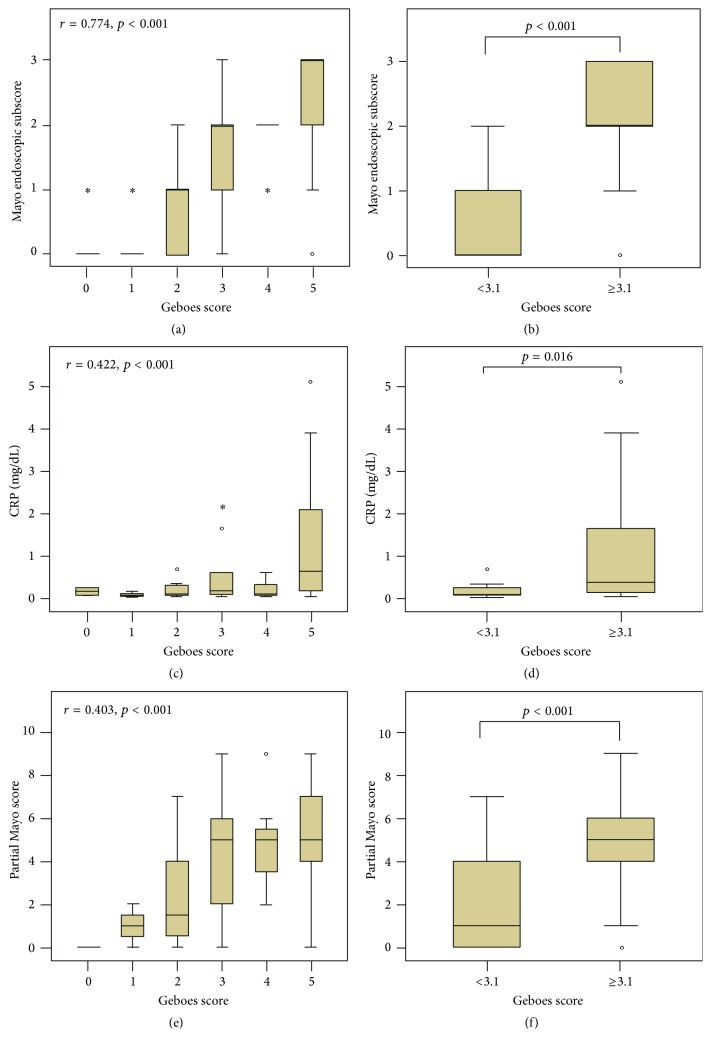
Correlation between histological activity (Geboes score) and endoscopic activity (Mayo endoscopic subscore), clinical activity (partial Mayo score), and serologic activity (CRP) in patients with UC.

A total of 154 biopsy specimens from 102 endoscopies in 82 UC patients were analyzed in this study. The histological activity index was closely correlated with the endoscopic activity index (*r* = 0.774, *p* < 0.001), clinical activity index (*r* = 0.403, *p* < 0.001), and CRP levels (*r* = 0.422, *p* < 0.001).  Figure 1 shows the proportion of histological scores according to the endoscopic scores. When the histological activities were divided according to Geboes score into inactive (<3.1) and active (≥3.1) disease, the endoscopic activity index, clinical activity index, and CRP levels were significantly higher in patients with active histological disease, compared to those with inactive histological findings.

**Figure 2:**
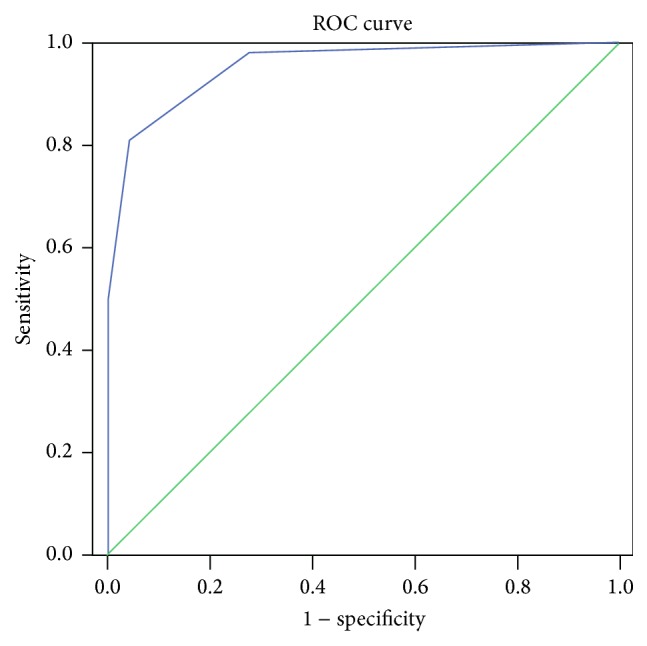
Receiver operator characteristics (ROC) curves of Mayo endoscopic subscore for the detection of histologically active disease.

To separate histologically active or inactive disease, the optimal cut-off of Mayo endoscopic subscore was found to be a score of 1. This provided an estimated area under the curve of 0.953 ± 0.016 (95% confidence interval: 0.921–0.986, *p* < 0.001), a sensitivity of 81.1%, and a specificity of 95.3% (Figure 2).

### 3.3. Histological Activity according to the Endoscopic Activity

We examined whether there were differences in the histological activity according to the endoscopic activity. Most of the mucosal samples in endoscopically moderate-to-severe disease (Mayo endoscopic subscores of 2-3) exhibited active histological inflammation (Geboes score of ≥3.1). However, some mucosal samples (21 of 62 samples) with mild or normal endoscopic findings exhibited evidence of histologically active disease. Furthermore, 66% (19 of 29 samples) of mucosa with endoscopically mild disease exhibited active histological inflammation (Table 3).

## 4. Discussion

To accurately monitor intestinal inflammation, a combination of clinical examinations, serology, endoscopy, and histology are needed for patients with UC. However, none of these monitoring methods is absolutely accurate, and each has its own strengths and weaknesses. Therefore, it is desirable to determine the relationship between the disease activity indices in clinical practice. Several studies have already demonstrated that endoscopic findings are correlated with clinical and serologic disease activities [14–17]. In addition, CRP levels are reportedly associated with the clinical, endoscopic, and histological disease activities in patients with inflammatory bowel disease (primarily Crohn's disease (CD)) [18]. However, few studies have assessed the correlation between histological activity and the endoscopic, clinical, and serologic activities in patients with UC.

Our results indicate that histological activity was most closely correlated with endoscopic activity in patients with UC. In particular, most mucosal samples with endoscopically moderate-to-severe disease exhibited active histological inflammation (Geboes score of ≥3.1), while most (94%) endoscopically normal mucosal samples exhibited a Geboes score of <3.1. One previous study of 378 colon sites in 54 UC patients reported that 88.1% (193 of 232) of the sites with a Mayo endoscopic subscore of 0 exhibited a histological score of 1, indicating inactive chronic colitis [16]. That finding is consistent with our results, although their histological score was not graded using the Geboes scoring system. In addition, we found a significant correlation between the Mayo endoscopic subscore and histological activity, with excellent specificity and sensitivity for a Mayo endoscopic subscore cut-off value of 1. Interestingly, despite the significant correlation, we observed histological inflammation in 66% of the mucosal samples with endoscopically mild disease, which has been considered mucosal healing in previous randomized controlled trials [19, 20].

This discrepancy can presumably be explained by the subjective nature of endoscopic findings. According to one previous study, interobserver variation for endoscopic assessment was greater in cases of normal or mild activity, compared to that in cases of moderate-to-severe activity [21]. In particular, erythema and vascular patterns (a component of endoscopically mild disease, Mayo endoscopic subscore of 1) exhibited relatively poor interobserver agreement, compared to erosion and ulceration (a component of moderate-to-severe disease, Mayo endoscopic subscore of 2-3) [21, 22]. Moreover, the ability of histologic assessment to see mild activity is much higher than that of endoscopy. We observed active histological inflammation in endoscopically normal mucosa (6%, 2 of 33 samples), which indicates that histological assessment may be able to more accurately evaluate UC disease activity, especially in endoscopically mild disease.

Our analysis revealed that the Geboes score was correlated with CRP levels in patients with UC. Among these patients, CRP is one of the most commonly used serologic parameters to assess intestinal inflammation [23]. However, given the inconvenience of endoscopy, several serologic markers have been evaluated, although CRP has exhibited good correlation with clinical and endoscopic activity in several studies [15, 18, 24]. Unfortunately, previous studies regarding the correlation between CRP levels and histological activity have reported conflicting results [16, 18]. In our study, CRP levels exhibited relatively good correlation with histological activity in patients with UC, although many patients with normal CRP levels exhibited active histological inflammation in their biopsy specimens. Interestingly, older studies regarding CRP levels and inflammatory bowel disease have reported that the CRP response was weaker in UC than that in CD [18, 25], which may be related to the fact that UC is a mucosal disease, whereas CD is a transmural disease. In addition, other investigators have reported that CRP levels did not differentiate between patients with and without endoscopic remission at five years after their UC diagnosis [26]. Therefore, these findings suggest that CRP levels may provide supportive information, although they are not sufficient to assess histological activity in UC.

The partial Mayo score is a noninvasive index of disease activity, which excludes the endoscopic appearance from the total Mayo score and is useful for assessing clinical severity and predicting the total Mayo score [27, 28]. In the present study, we observed a significant correlation between the Geboes and partial Mayo scores. However, in contrast to the Mayo endoscopic subscores, the partial Mayo scores were only weakly correlated with the Geboes scores. Previous studies have already reported a poor correlation between clinical symptoms and histological activity [12, 29], and there are several possible reasons for this correlation. First, a number of patients with UC remission have irritable bowel syndrome-like symptoms, despite minimal or no signs of inflammation [30–32]. Isgar et al. were the first to report the prevalence (33%) of irritable bowel syndrome-like symptoms in UC with clinical remission [30], and a recent prospective study has reported similar results [32]. Second, histology tends to only represent a small portion of the lesion; therefore, the clinical presentation can be mild even if the histological activity is severe. Therefore, our finding of a significant correlation between the histological and clinical activities should be interpreted with caution, given the relatively small sample of patients who were in clinical remission.

There are several potential limitations of this study. First, this was a retrospective study, and therefore the variables such as number and site of biopsies might not be consistent. Second, colonoscopy or sigmoidoscopy was mainly performed in patients with exacerbated or severe symptoms, rather than in asymptomatic patients. Thus, only a limited number of patients who had clinical remission or mucosal healing were enrolled, and selection bias cannot be excluded. Third, we did not check the fecal calprotectin, which is the promising fecal biomarker that reflects disease activity in UC [14, 33, 34]. Further study on the correlation with histological activity and fecal calprotectin will be needed. In addition, we evaluated the histological activity using the Geboes scoring system and compared the results to the clinical and endoscopic activities obtained via the Mayo scoring system. Therefore, our results may not be consistent with the clinical, endoscopic, and histological activities that are reported using other indices. However, all data in the present study were collected consecutively, with the same standard approach, and both the Mayo and Geboes scoring systems are broadly used in prospective trials and clinical practice.

In conclusion, our results demonstrated that the histological activity exhibited strong correlation with the endoscopic activity and moderate correlation with the clinical and serologic activities in patients with UC. Although the endoscopic activity most accurately reflected the histological activity, several patients with mild or normal endoscopic findings exhibited active histological inflammation in their biopsy samples. Therefore, histological assessment should be considered in patients with UC, regardless of their endoscopic activity.

## Figures and Tables

**Table 1:** Geboes scores for assessing ulcerative colitis histological activity.

Grade 0: structural (architectural) changes	

Subgrades	
0.0	No abnormality
0.1	Mild abnormality
0.2	Mild or moderate diffuse or multifocal abnormalities
0.3	Severe diffuse or multifocal abnormalities

Grade 1: chronic inflammatory infiltrate	

Subgrades	
1.0	No increase
1.1	Mild but unequivocal increase
1.2	Moderate increase
1.3	Marked increase

Grade 2: lamina propria neutrophils and eosinophils	

2A: eosinophils	
2A.0	No increase
2A.1	Mild but unequivocal increase
2A.2	Moderate increase
2A.3	Marked increase
2B: neutrophils	
2B.0	None
2B.1	Mild but unequivocal increase
2B.2	Moderate increase
2B.3	Marked increase

Grade 3: neutrophils in epithelium	

3.0	None
3.1	<5% crypts involved
3.2	<50% crypts involved
3.3	>50% crypts involved

Grade 4: crypt destruction	

4.0	None
4.1	Probable-local excess of neutrophils in part of the crypt
4.2	Probable-marked attenuation
4.3	Unequivocal crypt destruction

Grade 5: erosion or ulceration	

5.0	No erosion, ulceration, or granulation tissue
5.1	Recovering epithelium + adjacent inflammation
5.2	Probable erosion, focally stripped
5.3	Unequivocal erosion
5.4	Ulcer or granulation tissue

**Table 2:** Baseline patient characteristics (*n* = 82).

Number of endoscopies	102
Number of biopsies	154
Male (%)	49 (60%)
Age (years, mean ± SD)	47.5 ± 15.9
Duration of disease (years)	5.4 ± 5.4
Extent of UC (%)	
Proctitis	18 (22%)
Left-sided colitis	33 (40%)
Extensive colitis	31 (38%)
Medication at endoscopy (%)	
5-ASA	65 (64%)
Systemic corticosteroids	27 (26%)
Azathioprine	24 (24%)
TNF-alpha inhibitor	7 (7%)

SD: standard deviation, UC: ulcerative colitis, 5-ASA: 5-aminosalicylic acid, and TNF: tumor necrosis factor.

**Table 3:** Histological activity (Geboes score <3.1 and ≥3.1) according to the endoscopic activity (Mayo endoscopic subscore).

Histological activity	Endoscopic activity (Mayo endoscopic subscore)			
	0 (normal) *N* = 33	1 (mild) *N* = 29	2 (moderate) *N* = 37	3 (severe) *N* = 55
Geboes score < 3.1	31 (94%)	10 (34%)	2 (5%)	0 (0%)
Geboes score ≥ 3.1	2 (6%)	19 (66%)	35 (95%)	55 (100%)
